# Three Factors Are Critical in Order to Synthesize Intelligible Noise-Vocoded Japanese Speech

**DOI:** 10.3389/fpsyg.2016.00517

**Published:** 2016-04-26

**Authors:** Takuya Kishida, Yoshitaka Nakajima, Kazuo Ueda, Gerard B. Remijn

**Affiliations:** ^1^Human Science, Graduate School of Design, Kyushu UniversityFukuoka, Japan; ^2^Department of Human Science/Research Center for Applied Perceptual Science, Kyushu UniversityFukuoka, Japan

**Keywords:** speech perception, noise-vocoded speech, factor analysis, principal component analysis, critical band

## Abstract

Factor analysis (principal component analysis followed by varimax rotation) had shown that 3 common factors appear across 20 critical-band power fluctuations derived from spoken sentences of eight different languages [Ueda et al. (2010). Fechner Day 2010, Padua]. The present study investigated the contributions of such power-fluctuation factors to speech intelligibility. The method of factor analysis was modified to obtain factors suitable for resynthesizing speech sounds as 20-critical-band noise-vocoded speech. The resynthesized speech sounds were used for an intelligibility test. The modification of factor analysis ensured that the resynthesized speech sounds were not accompanied by a steady background noise caused by the data reduction procedure. Spoken sentences of British English, Japanese, and Mandarin Chinese were subjected to this modified analysis. Confirming the earlier analysis, indeed 3–4 factors were common to these languages. The number of power-fluctuation factors needed to make noise-vocoded speech intelligible was then examined. Critical-band power fluctuations of the Japanese spoken sentences were resynthesized from the obtained factors, resulting in noise-vocoded-speech stimuli, and the intelligibility of these speech stimuli was tested by 12 native Japanese speakers. Japanese mora (syllable-like phonological unit) identification performances were measured when the number of factors was 1–9. Statistically significant improvement in intelligibility was observed when the number of factors was increased stepwise up to 6. The 12 listeners identified 92.1% of the morae correctly on average in the 6-factor condition. The intelligibility improved sharply when the number of factors changed from 2 to 3. In this step, the cumulative contribution ratio of factors improved only by 10.6%, from 37.3 to 47.9%, but the average mora identification leaped from 6.9 to 69.2%. The results indicated that, if the number of factors is 3 or more, elementary linguistic information is preserved in such noise-vocoded speech.

## Introduction

It is important to understand what acoustic characteristics of speech sounds are essential for speech intelligibility in order to elucidate the cognitive mechanisms of speech communication. The acoustic characteristics of speech that contribute to speech perception have been investigated with many different approaches. One of the most fruitful methods is to control acoustic characteristics of speech by signal processing and then to test the intelligibility of the synthesized signals (for reviews see Diehl et al., 2004; Samuel, 2011). The temporal change of spectra is the representative acoustic characteristic in this context, and is processed by a frequency analyzer of the auditory system (Plomp, 1964; Plomp and Mimpen, 1968; Plack, 2013).

Perceptual experiments in which spectral information was systematically degraded revealed that perceptual cues embedded in speech spectra are highly redundant (Remez et al., 1981; Baer and Moore, 1993; Shannon et al., 1995; Warren et al., 1995). These studies often proceeded from the concept of auditory filters (Patterson, 1974; Moore, 2012) or critical bands (Fletcher, 1940), indicating parallel channels to process frequency components. Although the widths of the critical bands were determined from behavioral data, each of them corresponds to a distance of about 1.3 mm along the basilar membrane (Fastl and Zwicker, 2006). There are about 20 critical bands in the commonly used frequency range of speech sounds, which means that we can use the power fluctuations in these frequency bands to perceive speech. In most situations, however, we can perceive speech sounds represented by a relatively small number of power fluctuations because of the redundancy of perceptual cues in speech sounds. Shannon et al. (1995) found that four bands of amplitude-modulated noise were sufficient for nearly perfect scores (>95%) of word intelligibility. Many studies (e.g., Dorman et al., 1997; Loizou et al., 1999; Souza and Rosen, 2009; Ellermeier et al., 2015) have measured the intelligibility of noise-vocoded speech, and indicated results consistent with Shannon et al. (1995). These studies suggest that the 20 outputs of critical-band filters, for example, can be reduced to a smaller number of channels without sacrificing the speech intelligibility too much.

In the present study, the power fluctuations of speech signals in 20 critical-band filters were analyzed and resynthesized with a new method of factor analysis. This analysis method is a modification of principal component analysis followed by varimax rotation, and was developed to reduce the number of dimensions of observed variables while retaining the information conveyed by these variables as far as possible (Jolliffe, 2002).

One of the earliest studies that applied principal component analysis to speech sounds was conducted by Plomp et al. (1967). They found that 14 Dutch steady vowels were distinguishable on the first and second principal component plane; these first two principal components had a close relation with the first and the second formant of the vowels (Pols et al., 1973). Zahorian and Rothenberg (1981) performed principal component analyses of speech, and they suggested that 3–5 principal components might convey enough perceptual cues to make speech signals intelligible. In a more systematic study of Ueda et al. (2010), principal component analysis was followed by varimax rotation. They discovered that 3 common factors appeared in 20 critical-band power fluctuations derived from spoken sentences of eight different languages (American English, British English, Cantonese Chinese, French, German, Japanese, Mandarin Chinese, and Spanish). The same analysis was performed over speech samples from 15-, 20-, and 24-month-old infants, and the 3 common factors observed in adult voices were gradually formed along with language acquisition (Yamashita et al., 2013).

Thus, 3 factors seem to reflect an acoustic language universal, and these factors may play important roles in speech perception. This speculation, however, was brought about only from observations of acoustic characteristics of speech sounds, and it was not yet clear whether the extracted factors convey any perceptual cues. In the present study, we therefore examined how many factors were needed to make speech signals sufficiently intelligible. If the first 3 factors would indeed make up a basic framework of speech perception, then speech sounds resynthesized from these factors should be intelligible enough. We thus performed a perceptual experiment employing resynthesized speech stimuli.

## Speech analysis

The purpose of this analysis was to obtain power-fluctuation factors suitable for resynthesizing speech sounds.

### Materials

Two-hundred speech sentences each spoken by five male native speakers of British English, 200 sentences each spoken by five male native speakers of Japanese, and 78 sentences^1^ each spoken by five male native speakers of Mandarin Chinese were used in the present analysis. These materials were selected from a commercial speech database (NTT-AT., 2002), recorded digitally (16-bit linear quantization and sampling frequency of 16000 Hz). The mean fundamental frequencies of the spoken sentences were 126 Hz (*SD* = 30 Hz) in British English, 136 Hz (*SD* = 31 Hz) in Japanese, and 164 Hz (*SD* = 38 Hz) in Mandarin Chinese. The three languages were chosen from the languages analyzed in the previous study of Ueda et al. (2010) as representatives of different families of languages. These three languages have different linguistic rhythms; English is a stress-timed language, Japanese is a mora-timed language, and Mandarin Chinese is a syllable-timed language (Ramus et al., 1999).

### Procedure

Speech sentences were resampled every 1 ms with a 30-ms-long Hamming window. From the extracted short time segments, power spectra were obtained through a Fast Fourier Transformation (FFT). Following this, these power spectra were smoothed with a 5-ms shortpass lifter by cepstral analysis (for a review on cepstral analysis see Rabiner and Schafer, 1978) to remove unnecessary details of the spectra. A 5-ms shortpass lifter removed fine structure of the power spectra narrower than 200 Hz that reflected vocal folds vibrations. Smoothed power spectra were then divided into 20 critical bands, and averaged power was calculated for each band. Thus, 20 temporal power fluctuations were obtained. The 20 critical bandwidths were taken from Zwicker and Terhardt (1980). The bandwidths originally ranged from 0 to 6400 Hz, but since the range below 50 Hz is unrelated to speech, the 1st bandwidth was narrowed from [0–100 Hz] to [50–100 Hz] (Table 1).

**Table 1:** **Critical bands for analysis**.

**Band no**.	**Center frequency (Hz)**	**Passband (Hz)**
1	75	50–100
2	150	100–200
3	250	200–300
4	350	300–400
5	450	400–510
6	570	510–630
7	700	630–770
8	840	770–920
9	1000	920–1080
10	1170	1080–1270
11	1370	1270–1480
12	1600	1480–1720
13	1850	1720–2000
14	2150	2000–2320
15	2500	2320–2700
16	2900	2700–3150
17	3400	3150–3700
18	4000	3700–4400
19	4800	4400–5300
20	5800	5300–6400

*The center frequency (not necessarily the exact mathematical center) and cutoff frequencies were adopted from Zwicker and Terhardt (1980), except for the lowest band*.

**Figure 1:**
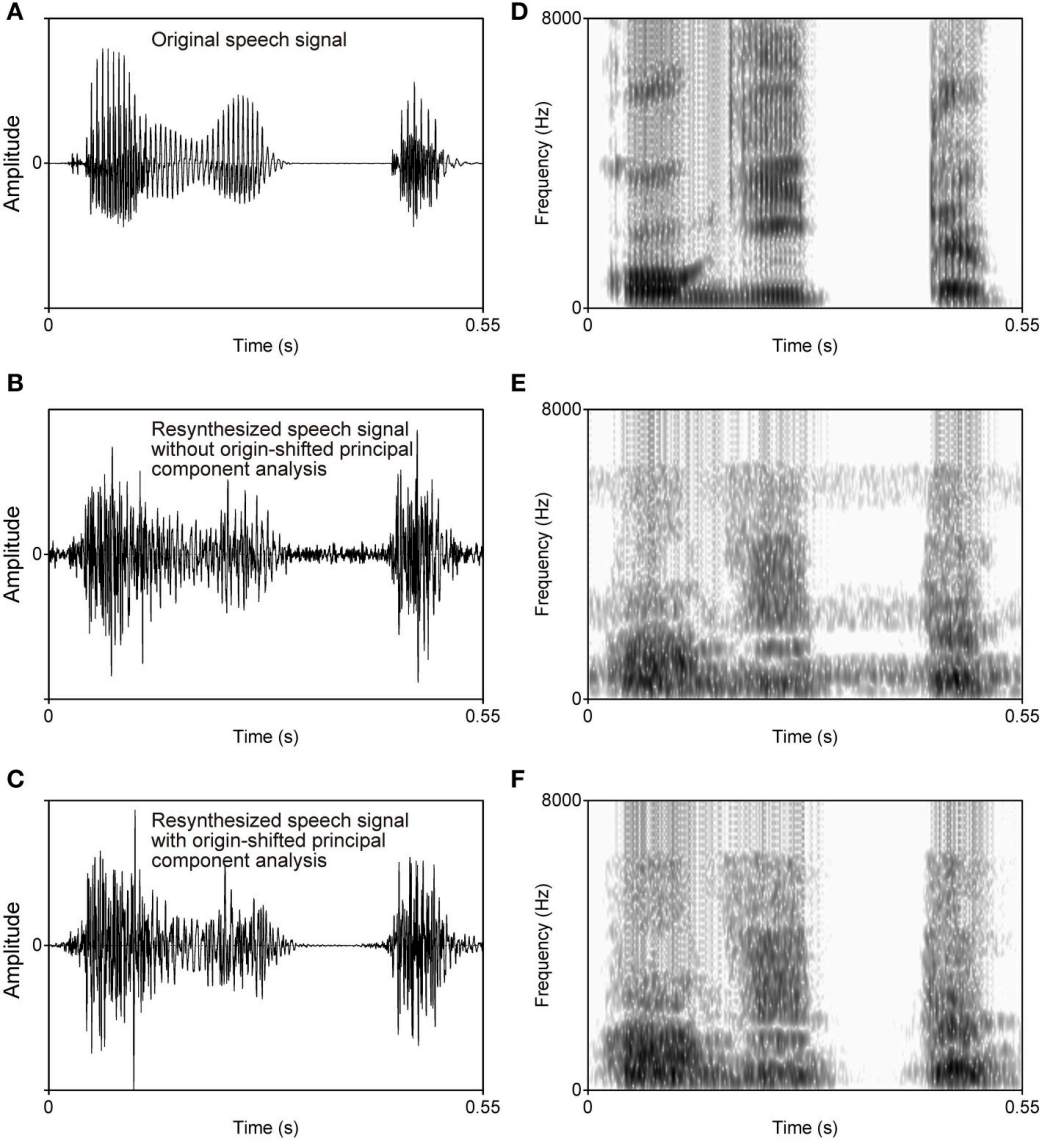
**Waveforms (left column) and spectrograms (right column) of an original speech signal (A,D), the resynthesized speech signal without origin-shifted principal component analysis (B,E), and the resynthesized speech signal with origin-shifted principal component analysis as proposed here (C,F)**. Steady background noise is observed in the speech signal resynthesized from the factors obtained with normal principal component analysis followed by varimax rotation **(B,E)**, but no such noise appears in the speech signal resynthesized from the factors obtained with origin-shifted principal component analysis followed by varimax rotation **(C,F)**.

The 20 power fluctuations were subjected to a new type of principal component analysis followed by varimax rotation. *Origin-shifted principal component analysis* as used in this study proceeds from the idea that calculated eigenvectors should originate not from the gravity center of the data but from the zero point^2^, i.e., acoustically silent point. If the silent point is not contained in the subspace of the principal components, resynthesized sounds should generate noise even at the point corresponding to the silent point. In other words, the silent point is mapped onto a point indicating a certain acoustic power. As a result, the listener perceives a steady background noise in the resynthesized speech sounds (probably, this kind of steady background noise should have appeared in Zahorian and Rothenberg's (1981) resynthesized speech). An example of a steady background noise in a resynthesized speech sound is shown in Figure 1.

The eigenvectors derived with origin-shifted principal component analysis were rotated by varimax rotation (Kaiser, 1958), resulting in power-fluctuation factors. The purpose of varimax rotation was to make the relation between the factors and the critical bands easier to interpret because the orthogonality of the factors was maintained. The total number of factors produced in the above procedure was varied from 1 to 9 (for example, when 3 power-fluctuation factors were obtained, the eigenvectors of the first 3 principal components were rotated).

### Results and discussion

**Figure 2:**
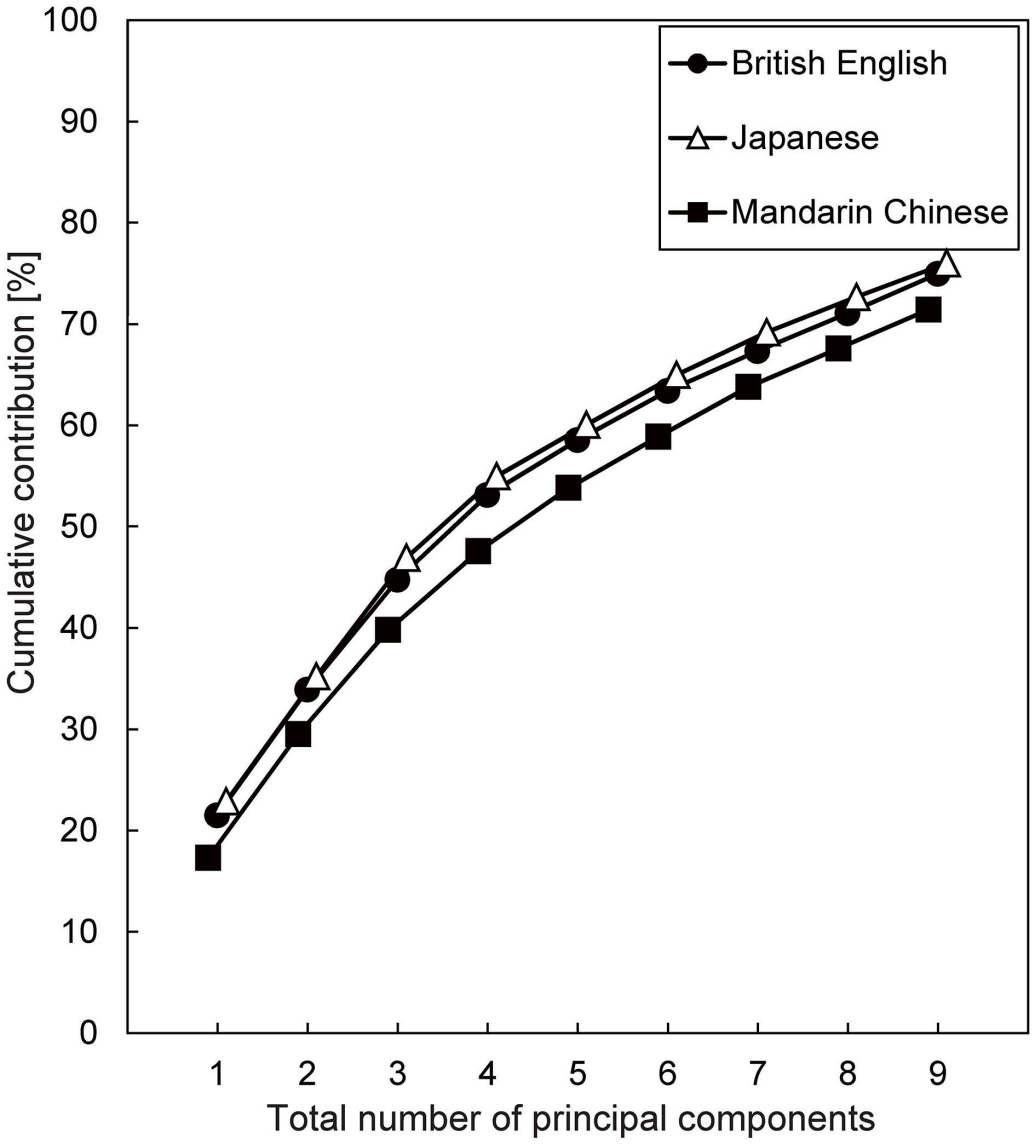
**The cumulative contribution as a function of the number of principal components for power fluctuations in the 20 critical-band filters**. Spoken sentences of three languages were investigated.

Figure 2 shows the cumulative contributions of the first 1–9 principal components. Over 70% variance of the power fluctuations was accounted for by the first 9 principal components in all three languages (75, 76, and 71% for British English, Japanese, and Mandarin Chinese, respectively). A plausible explanation for the lower cumulative contributions for Mandarin Chinese is that the mean fundamental frequency of Mandarin Chinese speech was higher than that of the other languages, and that the cepstral liftering could not smooth the power spectra sufficiently.

**Figure 3:**
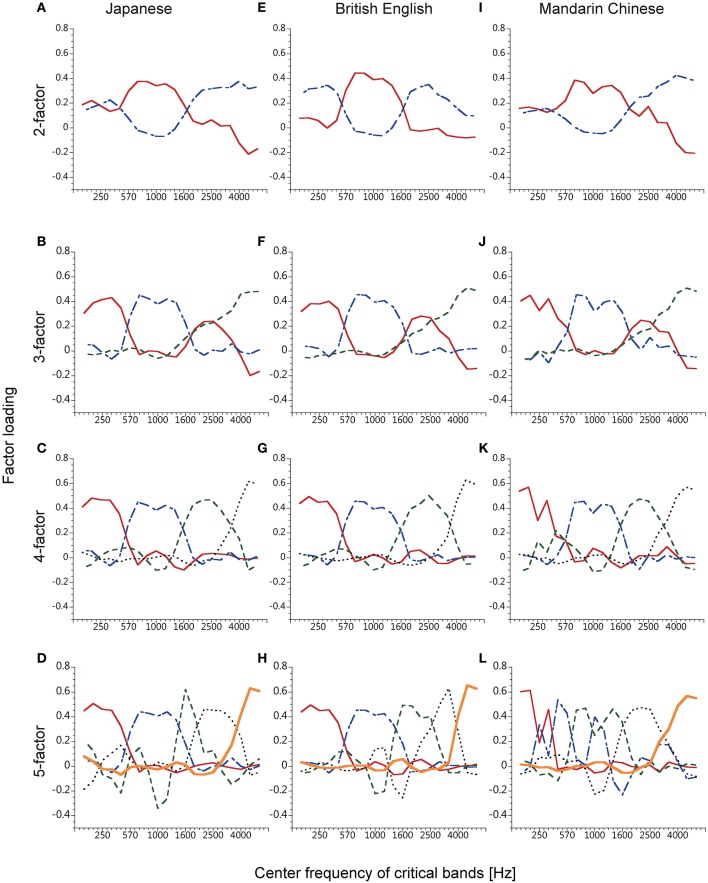
**Factor loadings of the factors obtained from the time series of smoothed speech spectra of five male speakers each of Japanese (A–D), British English (E–H), and Mandarin Chinese spoken sentences (I–L)**. The number of extracted factors was 2–5, from top to bottom.

Figure 3 shows factor loadings obtained with the three languages. The patterns of the power-fluctuation factors were similar among the three languages when the number of extracted factors was up to 4 (Figures 3A–C,E–G,I–K). The cumulative contributions of the 4 power-fluctuation factors were 53, 55, and 48% for British English, Japanese, and Mandarin Chinese, respectively. When the number of factors was 5 or larger, it was difficult to find similar patterns of factors among these languages (Figures 3D,H,L). This means that about 50% of variance in the 20 power fluctuations could be mapped onto a common subspace of 4 fluctuation factors for the three languages.

In the 3- and 4-factor analyses, the factors seemed to divide speech sounds into four frequency bands (about 50–550 Hz, 550–1700 Hz, 1700–3500 Hz, and over 3500 Hz). One of the factors obtained in the 3-factor analyses had high loadings at two frequency bands, the 1st and the 3rd band. These bimodal factors were first reported by Ueda and Nakajima (2008) and Ueda et al. (2010), in which they predicted that the bimodal factor would be separated into 2 factors if they could elaborate the analysis method. The predicted factors indeed appeared in the 4-factor analysis.

## Speech intelligibility experiment

The purpose of this experiment was to determine the number of power-fluctuation factors needed to make speech sufficiently intelligible. We chose Japanese speech sentences as the basis for sound stimuli. Japanese is convenient for scoring answers reported by participants because Japanese words can be broken up into morae, which are syllable-like phonological units. Each lexical mora is uniquely represented by a single Japanese “hiragana” letter used in writing.

### Participants

Six men and six women, ranging in age from 19 to 24 years old (mean age = 21.5 years, *SD* = 1.6 years), participated as volunteers. They were all native speakers of Japanese with pure-tone thresholds lower than 25 dB HL at audiometric frequencies of 125–8000 Hz for both ears. They were naive as to the purpose of the experiment. The procedure of the experiment was approved by the Ethics Committee of the Faculty of Design, Kyushu University. All participants provided written informed consent as to their participation.

### Equipment

The experiment was conducted in a sound-proof room, where the background noise level was below 25 dBA. The sound stimuli were generated digitally (16-bit linear quantization and sampling frequency of 16000 Hz), with a computer (Frontier KZFM71/N) equipped with an audio card (E-MU 0404). The sounds were presented binaurally (diotically) to the participant via a digital-to-analog converter (ONKYO, SE-U55GX), an active low-pass filter (NF DV-04 DV8FL, cutoff at 7000 Hz), a digital graphic equalizer (Roland, RDQ-2031), an amplifier (STAX, SRM-323S), and headphones (STAX, SR-307). The active low-pass filter was for avoiding aliasing, and the digital graphic equalizer was to equalize frequency responses of the headphones.

### Stimuli

Original speech signals for sound stimuli were digitally recorded Japanese sentences (16-bit linear quantization and sampling frequency of 16000 Hz), selected from a commercial speech database (NTT-AT., 2002). Fifty-seven sentences, each containing 17 to 19 morae (mean = 18 morae), spoken by a male speaker were used; nine sentences were used for training trials, three sentences for warm-up trials, and the remaining 45 sentences for measurement trials. These sentences were part of the 200 sentences used to determine the power-fluctuation factors in the analysis.

The original speech signals were resynthesized from factors as 20-band noise-vocoded speech. The number of factors was 1–9 resulting in nine conditions. The 45 sentences used for measurement trials were divided into nine lists, each containing five sentences of 17 to 19 morae (mean = 18 morae). Each list was assigned to a different factor-number condition, and the assignment of the sentence lists to the factor-number conditions was different among participants (Table 2). The nine sentences for training trials were also assigned to different factor-number conditions, but the assignment of sentences to the conditions was the same among participants. The three sentences used for warm-up trials were of 7- to 9-factor conditions.

**Table 2:** **The assignment of sentence lists to the factor number conditions for each participant**.

	**Sentence list**								
	**A**	**B**	**C**	**D**	**E**	**F**	**G**	**H**	**I**
Participant I	1	2	3	4	5	6	7	8	9
Participant II	9	1	2	3	4	5	6	7	8
Participant III	8	9	1	2	3	4	5	6	7
Participant IV	7	8	9	1	2	3	4	5	6
Participant V	6	7	8	9	1	2	3	4	5
Participant VI	5	6	7	8	9	1	2	3	4
Participant VII	4	5	6	7	8	9	1	2	3
Participant VIII	3	4	5	6	7	8	9	1	2
Participant IX	2	3	4	5	6	7	8	9	1
Participant X	9	8	7	6	5	4	3	2	1
Participant XI	8	7	6	5	4	3	2	1	9
Participant XII	7	6	5	4	3	2	1	9	8

*The number from 1 to 9 indicates the factor number condition. Each sentence list consisted of five sentences containing 17–19 morae each*.

**Figure 4:**
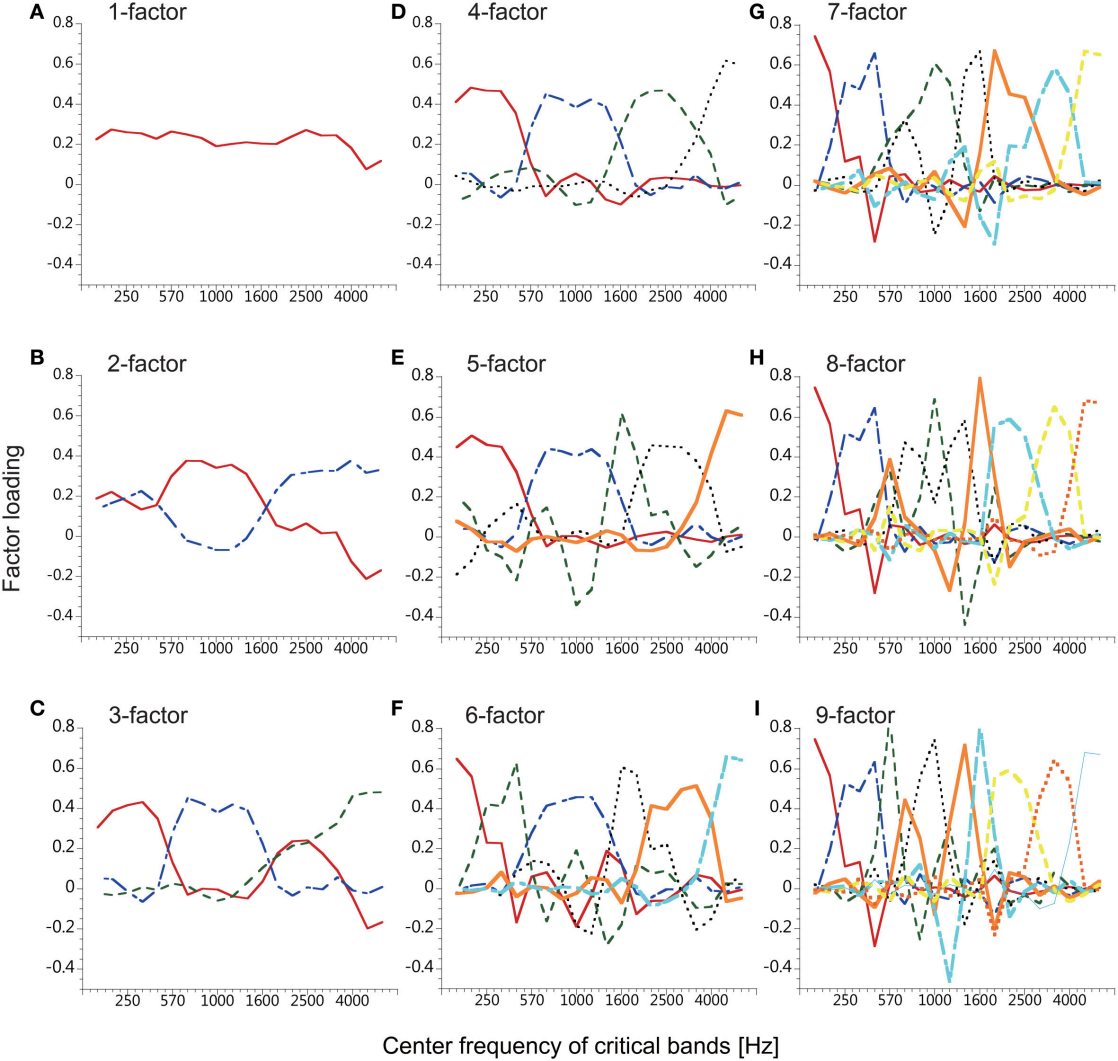
**Factor loadings of the factors used for resynthesis**. The factors were obtained from the time series of smoothed speech spectra of five male speakers of Japanese. The number of factors was 1–9 **(A–I)** resulting in nine conditions.

In order to synthesize noise-vocoded speech, the reproduced 20 power fluctuations of the original speech signal were used. With the same procedure as described in the Speech Analysis section, 20 power fluctuations were extracted from the original speech signal. To obtain time series of factor scores, the score of the *t*th time frame of the *k*th factor, *X*_*k, t*_ was calculated by the following equation:

(1) $$\begin{array}{r} X_{k,t}=\overset{20}{\underset{n=1}{\sum }}A_{k,n}Y_{n,t}, \end{array}$$

where *A*_*k, n*_ is the *n*th component of the normalized vector indicating the *k*th factor of the *K* (= 1…9) power-fluctuation factor(s) determined in the analysis of Japanese speech in the Speech Analysis section, and *Y*_*n, t*_ is the *t*th time frame of the power fluctuation in the *n*th critical band. *A*_*k, n*_ was different between the factor-number conditions as is plotted in Figure 4. Next, 20 power fluctuations were reproduced by

(2) $$\begin{array}{r} {Ŷ}_{n,t}=\overset{K}{\underset{k=1}{\sum }}A_{k,n}\;X_{k,t}, \end{array}$$

where, Ŷ_*n, t*_ is the *t*th time frame of the reproduced power fluctuation in the *n*th critical band. Geometrically, the transformations can be regarded as the projections of 20 power fluctuations in a 20-dimensional Euclidean space onto the *K*-dimensional subspace formed by the normalized vectors indicating the obtained factors.

White noise was generated, and was passed through banks of digital filters with the same cutoff frequencies as specified in Table 1. Twenty power fluctuations were then computed by squaring and smoothing each bandpass-filter output. The ratio between the reproduced power of the original speech signal as in equation (2) and the power of the generated noise was calculated in each critical band at each sample point^3^. The 20 bandpass-filtered noises were thus modulated with that ratio to realize the 20 reproduced power fluctuations of speech sound. Finally, the modulated bandpass-filtered noises were added up to yield noise-vocoded speech.

### Procedure

The intelligibility experiment started with one training block of nine trials, followed by three main blocks which each consisted of one warm-up trial and 15 measurement trials. The participant, who sat on a chair in front of the computer screen wearing headphones, was asked to click a “play” button on the screen for each trial. A sound stimulus was presented 0.5 s after the button was clicked. The presentation was repeated three times with 1.5-s intervals. After listening to the sound stimulus, the participant typed the morae (syllable-like phonological units) which he/she heard using hiraganas (Japanese moraic phonograms). The participant was instructed to avoid guessing parts of sentences which were not heard clearly. All stimuli in main blocks were presented in random order.

### Results and discussion

**Figure 5:**
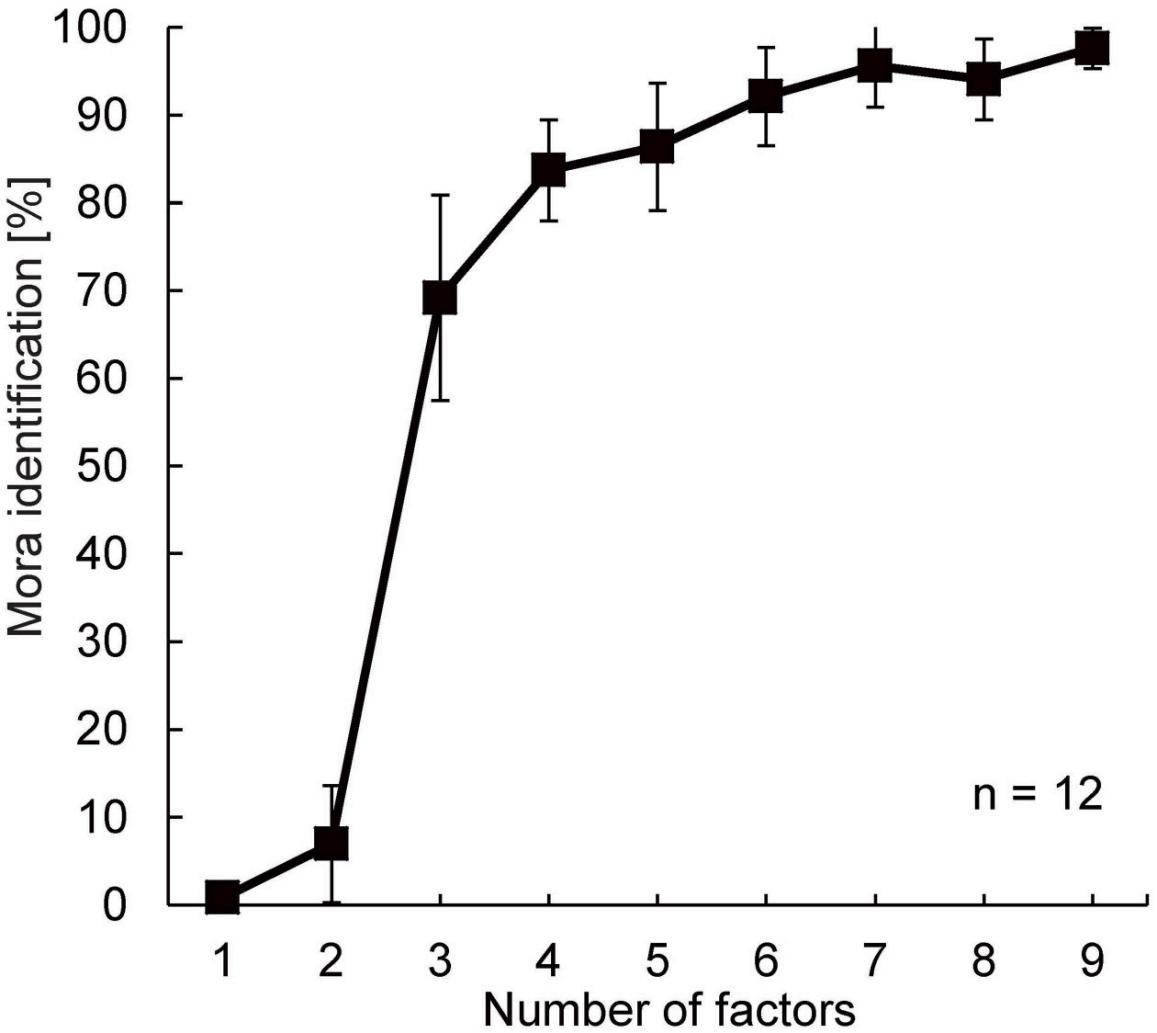
**Results of the speech intelligibility experiment**. The x-axis indicates the number of factors employed for reconstructing the 20 power fluctuations of the original speech sounds. The y-axis indicates mora identification. Error bars show standard deviations.

Figure 5 shows the percentage of mora identification as a function of the number of factors used to reconstruct the 20 power fluctuations of the Japanese speech stimuli. Mora identification increased with the number of factors, and approached a plateau around the 4-factor condition, where the participants' performance was 83.7% (*SD* = 5.8%). Mora identification was subjected to arcsine transformation, and a one-way Analysis of Variance (ANOVA) with repeated measures was performed. The results showed that the main effect of number of factors was significant [*F*_(8, 88)_ = 315.44, *p* < 0.0001]. *Post-hoc* tests according to Scheffe showed no statistically significant differences in mora identification when the number of factors was increased beyond 6 [*F*_(8, 99)_ = 3.83, *p* = 0.869, n.s.]. There was a significant difference [*F*_(8, 99)_ = 317.36, *p* < 0.001] in the mora identification between the 2- and the 3- [or more] factor conditions, and between the 3- and the 4- [or more] factor conditions [*F*_(8, 99)_ = 16.68, *p* < 0.05]. There was no significant difference between the mora identifications obtained with the 4- and the 5- factor condition [*F*_(8, 99)_ = 1.37, *p* = 0.994, n.s.], between the 4- and the 6-factor condition [*F*_(8, 99)_ = 11.09, *p* = 0.212, n.s.], or between the 5- and the 8- factor condition [*F*_(8, 99)_ = 10.15, *p* = 0.269, n.s.]. A remarkable improvement of mora identification appeared when the number of factors was changed from 2 to 3; the average mora identification leaped from 7% to about 70% [exactly from 6.9% (*SD* = 6.7%) to 69.2% (*SD* = 11.7%)]. The first 3 factors turned out to be critical for speech intelligibility.

## General discussion

We applied factor analysis to spoken sentences in three different languages, and performed an intelligibility test of Japanese sentences to investigate how power-fluctuation factors contributed to speech perception. The method of the factor analysis used in a previous study (Ueda et al., 2010) was modified in order to make it possible to resynthesize power fluctuations of speech across 20 critical bands from the obtained factors. The power-fluctuation factors extracted with this modified analysis method had very similar profiles to the ones in the previous studies (Ueda et al., 2010; Ellermeier et al., 2015). Twenty critical bands were divided into four frequency regions by the factors when the number of extracted factors was 3 or 4. These factors appeared commonly across three languages, i.e., British English, Japanese, and Mandarin Chinese. This was consistent with the results of Ueda et al. (2010). The drastic modification of the analysis method did not distort the essential features of the factors. The advantage of the present modification was that the vectors indicating the factors originated from the acoustically silent point. The silent point mapped onto the extracted subspace generated no noise: Silent parts remained silent when resynthesized.

The set of the 3 power-fluctuation factors proved to play a vital role in making speech intelligible. Although the 3 factors explained only 47.9% of the power fluctuations in the original speech sentences, 69.2% (*SD* = 11.7%) of the morae in the Japanese sentences were conveyed perceptually through these factors. Less than a half of the physical variance thus is very likely to be more informative than the rest. The finding that the 6-factor condition finally led to an asymptotic performance suggests that the information in the 3–4 factors forms the basis of perceptual cues, but that it is not yet sufficient to carry phonological details.

Let us compare the participants' performance in this study with that in four previous studies (Shannon et al., 1995; Dorman et al., 1997; Souza and Rosen, 2009; Ellermeier et al., 2015), which investigated the relationship between the number of vocoding channels and sentence recognition. Four-channel noise-vocoded speech induced high intelligibility in these previous studies (95% correct score in Shannon et al., 1995; Dorman et al., 1997; Ellermeier et al., 2015, and 70% correct score in Souza and Rosen, 2009). These correct scores are not too far from those obtained in the 3- and the 4-factor condition in our experiment. Very probably, four-channel noise-vocoded speech of which bandwidths are determined by the 3 or 4 factors in the present paradigm will be intelligible as well (see Ellermeier et al., 2015). The reason why high recognition performances were obtained with four-channel noise-vocoded speech in the previous studies can be explained if we assume that the fundamental nature of speech sounds consists of 3 or 4 bands of power fluctuations, and originates from constraints of the size and structure of the human articulatory organs (Yamashita et al., 2013, Figure A3). This conjecture comes from the fact that 3–4 factors commonly appeared in three different languages, and speech perception seems to be based on the perceptual cues carried by the 3–4 power-fluctuation factors.

If the power-fluctuation factors obtained in this study play an essential role in human speech communication, some correspondence will be found between the factors and articulatory movements as well as brain activities related to speech communication. The present findings might contribute to the development of technology supporting speech communication on various occasions.

## Author contributions

TK and YN designed the study. TK collected and analyzed the data, and the other authors supported him occasionally. All the authors interpreted the results together. TK wrote the first draft, and all the authors improved the paper together. YN gave the final approval of the version to be published.

### Conflict of interest statement

The authors declare that the research was conducted in the absence of any commercial or financial relationships that could be construed as a potential conflict of interest.

## Footnotes

^1^The number of Mandarin Chinese sentences was smaller than that of British English sentences or Japanese sentences because speech files with technical problems in the recordings were excluded from the analysis.

^2^Only when the gravity center of the data is identical with the zero point, the eigenvectors in the usual sense originate from the zero point. To realize this special case, principal component analysis was performed on the power fluctuations connected with their sign-reversed counterparts.

^3^The sample size of the 20 reproduced power fluctuations was increased by repeating values to equate with the sample size of the power fluctuations of the bandpass-filtered noises.
